# A novel correction method for modelling parameter-driven autocorrelated time series with count outcome

**DOI:** 10.1186/s12889-024-18382-4

**Published:** 2024-03-27

**Authors:** Xiao-Han Xu, Zi-Shu Zhan, Chen Shi, Ting Xiao, Chun-Quan Ou

**Affiliations:** State Key Laboratory of Organ Failure Research, Department of Biostatistics, School of Public Health, Southern Medical University, Guangzhou, 510515 China

**Keywords:** Autocorrelated count data, Parameter-driven models, Type I error inflation, Unbiased correction, Interrupted time-series

## Abstract

**Background:**

Count time series (e.g., daily deaths) are a very common type of data in environmental health research. The series is generally autocorrelated, while the widely used generalized linear model is based on the assumption of independent outcomes. None of the existing methods for modelling parameter-driven count time series can obtain consistent and reliable standard error of parameter estimates, causing potential inflation of type I error rate.

**Methods:**

We proposed a new maximum significant *ρ* correction (MSRC) method that utilizes information of significant autocorrelation coefficient *ρ* estimate within 5 orders by moment estimation. A Monte Carlo simulation was conducted to evaluate and compare the finite sample performance of the MSRC and classical unbiased correction (UB-corrected) method. We demonstrated a real-data analysis for assessing the effect of drunk driving regulations on the incidence of road traffic injuries (RTIs) using MSRC in Shenzhen, China. Moreover, there is no previous paper assessing the time-varying intervention effect and considering autocorrelation based on daily data of RTIs.

**Results:**

Both methods had a small bias in the regression coefficients. The autocorrelation coefficient estimated by UB-corrected is slightly underestimated at high autocorrelation (≥ 0.6), leading to the inflation of the type I error rate. The new method well controlled the type I error rate when the sample size reached 340. Moreover, the power of MSRC increased with increasing sample size and effect size and decreasing nuisance parameters, and it approached UB-corrected when *ρ* was small (≤ 0.4), but became more reliable as autocorrelation increased further. The daily data of RTIs exhibited significant autocorrelation after controlling for potential confounding, and therefore the MSRC was preferable to the UB-corrected. The intervention contributed to a decrease in the incidence of RTIs by 8.34% (95% CI, -5.69–20.51%), 45.07% (95% CI, 25.86–59.30%) and 42.94% (95% CI, 9.56–64.00%) at 1, 3 and 5 years after the implementation of the intervention, respectively.

**Conclusions:**

The proposed MSRC method provides a reliable and consistent approach for modelling parameter-driven time series with autocorrelated count data. It offers improved estimation compared to existing methods. The strict drunk driving regulations can reduce the risk of RTIs.

**Supplementary Information:**

The online version contains supplementary material available at 10.1186/s12889-024-18382-4.

## Background

Count time-series data are a common type of data in environmental epidemiology and public health, such as monthly road traffic deaths [1] and daily hospital admissions [2]. These count data are generally autocorrelated since the series are measured sequentially over time [3]. Previous studies generally use the maximum likelihood estimate of the traditional generalized linear model (GLM) as the point estimate of the regression coefficients, which are consistent and asymptotically normal [4, 5]. However, the GLM is based on the assumption that the outcome observations are independent, which ignores the potential autocorrelation of the data and therefore leading to an underestimation of standard error, probably resulting in false positive results when estimating the effect of influencing factors [6].

The modelling of autocorrelated count data is complex, particularly as it has not yet been developed in a unified framework. Cox (1981) [7] proposed to divide the modelling methods into two categories: observation-driven model and parameter-driven model. In the observation-driven model, the autocorrelation is specified by directly incorporating the lagged values of observed counts (e.g., first-order autoregressive (AR(1))) into the mean function of the outcome. The regression parameters can be directly estimated by maximum likelihood estimation. However, the interpretation of the regression parameters may be challenging, since it represents the effect of corresponding covariates on the expectation of count outcomes conditional on the history of outcomes. For the parameter-driven model, the serial autocorrelation is driven by an unobserved latent process. That is, the parameter-driven model can be considered as GLM with a pre-specified dependence structure [8], allowing for straightforward interpretability of regression coefficients. However, the parameter estimation by maximizing the full likelihood function is also very difficult to perform the *n*-fold integral of the AR(1) Gaussian probability density function of the latent process [9]. Therefore, applied research has commonly not checked the problem of autocorrelation or disregarded this issue even if it exists, mainly because of methodological challenges. To attempt to resolve the methodological problem, Davis et al. (2000) [10] developed an unbiased correction (UB-corrected) method to obtain the asymptotic properties of the GLM estimator by adjusting the autocovariance matrix based on the nuisance parameters of the latent process. The estimates of the nuisance parameters, including the variance and the first-order autocorrelation coefficient *ρ*(1), are obtained from the moment estimation proposed by Zeger (1988) [11]. Although this method has raised many concerns [12–14], it still tends to underestimate the standard error (SE) in the presence of time-varying covariates, which can lead to the inflation of the type I error rate [13, 15].

A real data analysis was motivated by the issue of autocorrelation. In May 2011, China introduced the criminalization of drunk driving, followed by a series of detailed penalties to enhance its implementation. Road traffic injuries (RTIs), as a crucial indicator of road safety, are affected by drunk driving regulations. Previous studies have assessed the time-invariant intervention effect of these regulations based on annual or monthly data [16–18]. However, the effect of drunk driving regulations is most likely to vary over time due to the successive introduction and increased enforcement of related regulations. Therefore, based on daily data of RTIs, the investigation of the potential time-varying effects is of great significance for modification and generalization of the intervention. In addition, only one previous study considered the potential autocorrelation of the RTIs. It addressed the issue by using an observation-driven model, the autoregressive integrated moving average model after transforming the count data into rates, which might ignore the specific distribution and dispersion of the count data [17]. Meanwhile, our preliminary analyses showed that the RTIs data still presented a significant autocorrelation after controlling for seasonality, long-term trend and meteorological factors. It is necessary to develop an appropriate parameter-driven method.

To ensure effective statistical inference on the regression coefficients, it is essential to address the issue of underestimation in the SE. Therefore, on the basis of the UB-corrected method, this study innovatively proposed a new correction method using the maximum of the significant *ρ* estimates within order 5. A Monte Carlo simulation was used to evaluate and compare the finite sample performance between the UB-corrected method and the new method. Then, the intervention effect of drunk driving regulations on RTIs was assessed to demonstrate the application of the proposed method. This study may provide some insights into the method development and practical application of the parameter-driven model of autocorrelated time series with count data.

## Methods

### Parameter-driven autocorrelated time series of count data

In this study, the time series of count observed at time *t* is denoted as {${Y}_{t}$: *t* = 1, …, *n*}. When the counting process is assumed to follow a Poisson distribution, the specific form is as follows:

$${Y}_{t}\mid {\alpha }_{t},{\varvec{x}}_{t}\sim Poisson\left(\text{e}\text{x}\text{p}({\alpha }_{t}+{\varvec{x}}_{\varvec{t}}^{\text{T}}\varvec{\beta })\right),$$

where ${\varvec{x}}_{\varvec{t}}$ is the *p*-dimensional regressors; $\varvec{\beta }$ is the corresponding vector of coefficients; and ${\alpha }_{t}$ is a latent process following its stochastic mechanism, which is usually assumed to follow a stationary Gaussian process with mean ${\mu }_{\alpha }$, variance ${\sigma }_{\alpha }^{2}$, autocovariance function $\gamma_{\alpha }\left(h\right)$, and autocorrelation function ${\rho }_{\alpha }\left(h\right)$ [9, 10, 19]. To satisfy the identifiability, it is necessary to make E($\text{e}\text{x}\text{p}\left({\alpha }_{t}\right)$) = 1, i.e., ${\alpha }_{t}\sim{N}(-{\sigma }_{\alpha }^{2}/2{,\sigma }_{\alpha }^{2})$. The marginal mean, variance, and autocorrelation forms of ${Y}_{t}$ are as follows [12]:

$$\text{E}\left[{Y}_{t}\right]={\mu }_{t}=\text{exp}\left({\varvec{x}}_{\varvec{t}}^{\text{T}}\varvec{\beta }\right),$$

$$\text{V}\text{a}\text{r}\left[{Y}_{\text{t}}\right]={\mu }_{t}+{\sigma }_{w}^{2}{\mu }_{t}^{2},$$

$${\rho }_{Y}\left(h\right)=\text{Corr}\left({Y}_{t},{Y}_{t+h}\right)=\frac{{\rho }_{w}\left(h\right)}{{\left\{1+{\left({\sigma }_{w}^{2}{\mu }_{t}\right)}^{-1}\right\}}^{\frac{1}{2}}{\left\{1+{\left({\sigma }_{w}^{2}{\mu }_{t+h}\right)}^{-1}\right\}}^{\frac{1}{2}}},$$

where ${w}_{t}=\text{e}\text{x}\text{p}\left({\alpha }_{t}\right)$, ${\sigma }_{w}^{2}=\text{exp}\left({\sigma }_{\alpha }^{2}\right)-1$, and ${\rho }_{w}\left(h\right)=\frac{\text{e}\text{x}\text{p}\left({\rho }_{\alpha }\left(h\right){\sigma }_{\alpha }^{2}\right)-1}{\text{exp}\left({\sigma }_{\alpha }^{2}\right)-1}$ with *h* ≥ 0. The outcomes are unconditionally correlated due to the serial correlation of the latent process, and the autocorrelation increases with the nuisance parameters ${\sigma }_{w}^{2}$ and ${\rho }_{w}\left(h\right)$.

### GLM estimator with UB-corrected covariance

The GLM can be used to estimate the parameters by ignoring the latent process, and the resulting regression coefficients ***β*** are consistent and asymptotically normal. However, the estimation of the covariance matrix must take into account the impact of the latent process [10]:

$$\mathrm{Var}\left({\hat{\boldsymbol\beta}}_{\mathrm{GLM}}\right)=\hat{\mathrm\Omega}_{I,n}^{-1}+\hat{\mathrm\Omega}_{I,n}^{-1}{\hat{\mathrm\Omega}}_{II,n}\hat{\mathrm\Omega}_{I,n}^{-1},$$

$${\hat{\mathrm\Omega}}_{I,n}=\sum\nolimits_{t=1}^n{\boldsymbol x}_{\boldsymbol t}\boldsymbol x_{\boldsymbol t}^\text{T}\text{exp}\left(\boldsymbol x_{\boldsymbol t}^\text{T}{\hat{\boldsymbol\beta}}_{\text{G}\text{L}\text{M}}\right),{\hat{\mathrm\Omega}}_{II,n}=\sum\nolimits_{t=1}^n\sum\nolimits_{s=1}^n{\boldsymbol x}_{\mathbf t}\boldsymbol x_{\boldsymbol s}^\text{T}\text{e}\text{x}\text{p}\left(\left(\boldsymbol x_{\boldsymbol t}^\text{T}+\boldsymbol x_{\boldsymbol s}^\text{T}\right){\hat{\boldsymbol\beta}}_{\text{G}\text{L}\text{M}}\right){\hat{\gamma}}_w(s-t),$$

where ${\hat{\Omega}}_{I,n}^{-1}$ is the estimate of the asymptotic covariance matrix obtained from the standard GLM, ${\hat{\Omega}}_{I,n}^{-1}{\hat{\Omega}}_{II,n}{\hat{\Omega}}_{I,n}^{-1}$ is the additional covariance imposed by the presence of the latent process, and ${\gamma }_{w}\left(s-t\right)$ is the autocovariance function of process ${w}_{t}$. When the latent process does not exist, i.e., ${\hat{\gamma }}_{w}\left(s-t\right)=0$ and ${\hat{\Omega}}_{II,n}=0$, the model degenerates into a classical GLM.

The estimation of ${\gamma }_{w}$ depends on the nuisance parameters ${{\sigma }_{w}}^{2}$ and ${\rho }_{w}$, which would be underestimated by directly using the GLM estimates ${\hat{\mu }}_{t}$. Therefore, Davis et al. (2000) [10] proposed UB-corrected estimates of nuisance parameters using the asymptotic unbiased estimator ${\mu }_{t}^{2}$ as ${\hat{\mu }}_{t}^{2}\text{e}\text{x}\text{p}(-2{\varvec{x}}_{\varvec{t}}^{\text{T}}{\hat{G}}_{n}{\varvec{x}}_{\varvec{t}})$, where ${\hat{G}}_{n}={\hat{{\Omega }}}_{I,n}^{-1}+{\hat{{\Omega }}}_{I,n}^{-1}{\hat{{\Omega }}}_{II,n}{\hat{{\Omega }}}_{I,n}^{-1}$ is the asymptotic covariance matrix. The specified form of the correction is as follows:

$${\hat{\sigma}}_{w,UB}^{2}=\frac{\sum\nolimits_{t=1}^{n} \left\{{\left({Y}_{t}-{\hat{\mu }}_{t}\right)}^{2}+{\hat{\mu}}_{t}^{2}\exp(-2{\varvec{x}}_{\varvec{t}}^{\text{T}}{\hat{G}}_{n}{\varvec{x}}_{\varvec{t}})\left(\exp\left(2{\varvec{x}}_{\varvec{t}}^{\text{T}}{\hat{G}}_{n}{\varvec{x}}_{\varvec{t}}\right)-2\exp({\varvec{x}}_{\varvec{t}}^{\text{T}}{\hat{G}}_{n}{\varvec{x}}_{\varvec{t}}/2)+1\right)-{\hat{\mu}}_{t}\right\}}{\sum\nolimits_{t=1}^{n}{\hat{\mu}}_{t}^{2}\text{e}\text{x}\text{p}\left(-2{\varvec{x}}_{\varvec{t}}^{\text{T}}{\hat{G}}_{n}{\varvec{x}}_{\varvec{t}}\right)},$$

$${\hat{\rho }}_{w,UB}\left(h\right)={\left({\hat{\sigma }}_{w,UB}^{2}\right)}^{-1}{\left(\sum_{t=1}^{n-h}{\hat{\mu }}_{t} {\hat{\mu }}_{t+h}{g}_{t,h}\right)}^{-1}\sum\nolimits_{t=1}^{n-h}\left\{\left({Y}_{t}-{\hat{\mu }}_{t}\right)\left({Y}_{t+h}-{\hat{\mu }}_{t+h}\right)+{\hat{\mu }}_{t}{\hat{\mu }}_{t+h}{g}_{t,h}\left(1-\text{e}\text{x}\text{p}\left({\varvec{x}}_{\varvec{t}}^{\text{T}}{\hat{G}}_{n}{\varvec{x}}_{\varvec{t}}/2\right)-\text{e}\text{x}\text{p}({\varvec{x}}_{\varvec{t}+\varvec{h}}^{\text{T}}{\hat{G}}_{n}{\varvec{x}}_{\varvec{t}+\varvec{h}}/2)+1/{g}_{t,h}\right)\right\},$$

where ${\hat{{\Omega }}}_{II,n}=\sum\nolimits_{h=-L}^{L} \sum\nolimits_{t=\text{m}\text{a}\text{x}(1-h,1)}^{\text{m}\text{i}\text{n}(n-h,n)} {\varvec{x}}_{\varvec{t}}{\varvec{x}}_{\varvec{t}+\varvec{h}}^{\text{T}}{\hat{\mu }}_{t}{\hat{\mu }}_{t+h}{\hat{\gamma }}_{w}\left(h\right)$, and the maximum lag *L* is used to better approximate the infinite series [10]. In addition, ${g}_{t,h}=\text{e}\text{x}\text{p}\left\{-{\left({\varvec{x}}_{\varvec{t}}+{\varvec{x}}_{\varvec{t}+\varvec{h}}\right)}^{\text{T}}{\hat{G}}_{n}\left({\varvec{x}}_{\varvec{t}}+{\varvec{x}}_{\varvec{t}+\varvec{h}}\right)/2\right\}$. The estimated autocorrelation parameters vary under different orders, and the estimation of parameter *ρ* at $h=1$ is treated as the true value in practice [10, 14]. However, Wu (2012) [19] showed that although UB-corrected provides a consistent estimator for nuisance parameters, it is problematic to obtain the asymptotic covariance matrix estimation, leading to the inflation of the type I error rate.

### A new method: maximum significant ρ correction (MSRC)

In order to solve the issue of underestimation of the covariance matrix, we attempted to adjust the estimate of ${\rho }_{w,UB}$. Previous studies have demonstrated that the choice of order is crucial and that a fixed autocorrelation order may not always be an optimal measure [20, 21]. The time series with higher autocorrelation may need a higher order to estimate the autocorrelation matrix [22]. Therefore, instead of solely using the autocorrelation estimate of order 1 as the true value, we fully utilized the information of multiple significant orders. First, autocorrelation coefficients for different orders were tested using the following Z test:

$$Z=\frac{{\hat{\rho }}_{w,UB}\left(h\right)}{\widehat{V}\left\{{\hat{\rho }}_{w,UB}\left(h\right)\right\}},$$

where $\widehat{V}\left\{{\hat{\rho }}_{w,UB}\left(h\right)\right\}={\left(\sum\nolimits_{t=1}^{n-h}{\hat{\mu }}_{t} {\hat{\mu }}_{t+h}\right)}^{-2}\sum\nolimits_{t=1}^{n-h}{\hat{\mu }}_{t}^{2}{\hat{\mu }}_{t+h}^{2}\left(1+{\hat{\mu }}_{t}^{-1}{\hat{\sigma }}_{w,UB}^{-2}\right)\left(1+{\hat{\mu }}_{t+h}^{-1}{\hat{\sigma }}_{w,UB}^{-2}\right)$, and the significance level of the autocorrelation test was set at 0.01 [22].

Then, multiple estimates of ${\rho }_{w,UB}\left(1\right)$ can be obtained by transforming ${\rho }_{w,UB}\left(h\right)$ for different orders. Taking the common AR(1) parameter-driven model as an example, its autocorrelation matrix is as follows:

$$\begin{pmatrix}1&\rho&\rho^2&\rho^3&\cdots\\ \rho&1&\rho&\rho^2&\cdots\\ \rho^2&\rho&1&\rho&\cdots\\ \vdots&\vdots&\vdots&\vdots&\cdots\\ &\cdots&&\rho&1\end{pmatrix}.$$

The ${\rho }_{w,UB}\left(h\right)$ needs to take the square root of the corresponding order to obtain multiple estimates. The existing literature of moment estimation emphasized the importance of considering higher-order information but did not demonstrate how to specify the optimal number of orders. Wang et al. (2012) [23] showed the necessity of using 5 lags of the partial autocorrelation function of the residuals based on influenza-associated mortality data. In this study, to avoid over-utilizing the information of high orders to affect the robustness of the estimation, we chose the maximum autocorrelation coefficients within the first 5 orders as the autocorrelation estimate. It is expected that a more conservative estimate of the covariance matrix with control over the type I error rate can be obtained by the new estimator (denoted as ${\rho }_{w,MSRC}$). Meanwhile, we performed a sensitivity study using different orders of moment estimation.

### Simulation study

We performed a Monte Carlo simulation to evaluate the finite sample performance of the MSRC method. The simulation parameters were specified based on the interrupted time-series (ITS) model which has been widely used for intervention evaluation [24]. In detail, the count time series was assumed to follow a Poisson distribution $Poisson\left(\text{e}\text{x}\text{p}({\alpha }_{t}+{\varvec{x}}_{\varvec{t}}^{\text{T}}\varvec{\beta })\right)$. The latent process ${\alpha }_{t}$ was assumed to follow the correlation structure of a Gaussian AR(1) process, with a variance ${\sigma }_{\alpha }^{2}$ of 0.5 and 1.0 and an autocorrelation coefficient ${\rho }_{\alpha }$ of 0.2, 0.4, 0.6, and 0.8, respectively. The covariate matrix was ${\varvec{x}}_{\varvec{t}}^{\text{T}}={\left(1,t,X,X(t-{t}_{0})\right)}^{\text{T}}$, and the corresponding true regression coefficient was $\varvec{\beta }={\left(\beta_{0},\beta_{t},{\beta }_{X},{\beta }_{X(t-{t}_{0})}\right)}^{\text{T}}$, where *t* = 1,2, …, *n* denoted a linear trend term; *X* represented the indicator variable of the intervention (0 for pre-intervention; 1 for post-intervention), ${t}_{0}$ denoted the starting time point of the intervention, $X(t-{t}_{0})$ represented the interaction term between the trend and the intervention variable, and the coefficients ${\beta }_{X}$ and ${\beta }_{X(t-{t}_{0})}$ indicated the level and trend change after the intervention, respectively. For simplicity, we assumed the number of time points before and after the intervention to be equal; hence, ${t}_{0}=n/2$ throughout the simulation study.

The statistical performances of the UB-corrected method and the new method were evaluated using the bias of the coefficients and the estimates of the nuisance parameters, type I error rate and statistical power. The type I error rate was examined for the simulated data with the true regression coefficient $\varvec{\beta }$ of ${\left(\text{1,1},\text{0,0}\right)}^{\text{T}},{ \left(\text{0.5,1},\text{0,0}\right)}^{\text{T}}$ and ${\left(\text{1,0.5,0},0\right)}^{\text{T}}$, respectively, to comprehensively assess the impact of the intercept and linear trend. For the sample size, we considered *n* = 20, 60, 100, …, 500. In addition, to compare the statistical power of the two methods (i.e., UB-corrected and MSRC), we considered three scenarios separately: (i) level change: $\varvec{\beta }={\left(\text{1,1},\text{0.4,0}\right)}^{\text{T}}$; (ii) trend change: ${\varvec{\beta }=\left(\text{1,1},\text{0,1.2}\right)}^{\text{T}}$; and (iii) both level and trend change: ${\varvec{\beta }=\left(\text{1,1},\text{0.4,1.2}\right)}^{\text{T}}$. Meanwhile, we set four scenarios ${\left(\text{1,1},\text{0.5,0}\right)}^{\text{T}}$, ${\left(\text{1,1},\text{0,1.5}\right)}^{\text{T}}$, ${\left(\text{1,1},\text{0.5,1.2}\right)}^{\text{T}}$ and ${\left(\text{1,1},\text{0.4,1.5}\right)}^{\text{T}}$ to fully evaluate the influence of effect size on power. Since our preliminary simulation revealed that the type I error rate was well controlled when the sample size was not less than 340, we set the sample size to *n* = 340, 360, 380, …, 500 for the investigation of power. The first two scenarios were examined using the Wald test. For the later scenarios, we used the covariance matrix to construct the test statistic ${\left({\hat{\beta }}_{X}, {\hat{\beta }}_{X(t-{t}_{0})}\right)}^{T}{\left[\text{V}\text{a}\text{r}\right({\hat{\beta }}_{X}, {\hat{\beta }}_{X(t-{t}_{0})}\left)\right]}^{-1}({\hat{\beta }}_{X}, {\hat{\beta }}_{X(t-{t}_{0})})$ for the intervention term, which follows a chi-square distribution with 2 degrees of freedom. In addition, we used the empirical standard error obtained from Monte Carlo simulation estimation to calculate the power. However, due to its unavailability in practice, this study only used the empirical power obtained from this estimation as a reference for comparison. Each simulation was repeated 10 000 times (Supplemental Code S1).

### Real data application

To assess the effect of the drunk driving intervention on RTIs, we obtained daily data on ambulance emergency call-outs for all RTIs from January 1, 2010, to December 31, 2016, from the Shenzhen pre-hospital care center. Based on the ITS design, we modelled daily RTIs ${Y}_{t}$ using a Poisson regression that adjusted for long-term and seasonal trends. Two meteorological factors (precipitation and temperature) were considered as covariates since previous studies have revealed their impacts on RTIs [25, 26]. The model formula was specified as follows:

$$\text{Log}\left(\text{E}\left[{Y}_{t}\right]\right)=\text{o}\text{f}\text{f}\text{s}\text{e}\text{t}\left(\text{L}\text{o}\text{g}\left(Pop*Car\right)\right)+{\beta }_{0}+\sum\nolimits_{\theta =1}^{k}\left[{\beta }_{1}\text{sin}\left(\frac{2\theta \pi t}{T}\right)+{\beta }_{2}\text{cos}\left(\frac{2\theta \pi t}{T}\right)\right]+{\beta }_{3}{Dow}_{t}+{\beta }_{4}{Holiday}_{t}+{\beta }_{5}{prec}_{t}+{\beta }_{6}ns\left({Temp}_{t},3\right)+{\beta }_{7}{X}_{t}+{\beta }_{8}t+{\beta }_{9}{X}_{t}\times ns(t,5),$$

where the population and motor vehicles were set as offsets, so that the fitted model was used to explore the impact factors of the incidence of RTIs [1]; the paired sine and cosine functions were used to fit the seasonality and *T* = 365.25; *k* = 2 was chosen by the mass spectrogram; ${Dow}_{t}$ is an indicator of the day of the week, ${Holiday}_{t}$ and ${prec}_{t}$ are categorical variables for public holidays (i.e., 0 for non-holiday, 1 for Chinese Spring Festival, and 2 for other holiday) and precipitation (i.e., none: 0.0 mm/h; mild: 0.0-2.5 mm/h; severe: >2.5 mm/h), respectively; *ns *(${Temp}_{t},3$) is a natural cubic spline of mean temperature with 3 degrees of freedom, which is chosen by the lowest Akaike’s Information Criterion. ${X}_{t}$ is the indicator variable of intervention, and ${X}_{t}\times ns(t,5)$ denotes the interaction term of the intervention variable and the spline function of time to fit the non-linear time-varying effect of the drunk driving intervention.

To better understand the public health significance of drunk driving regulations, we estimated the excess risk (ER) at time *t* by $(\text{exp}\left({\hat{\beta }}_{7}+{\hat{\beta }}_{9}\times ns(t,5)\right)-1)\times 100\%$, which represents the percentage change in the relative risk of the incidence of RTIs associated with the drunk driving intervention. Moreover, we provided estimates of ER at 3 time points: 1, 3, and 5 years after the intervention. The SE used in estimating the 95% confidence interval was calculated as $\sqrt{\left(1,ns(t,5)\right)\times \mathbf{V}\left({\hat{\beta }}_{7},{\hat{\beta }}_{9}\right)\times (1,{ns(t,5))}^{T}}$, noting that $\mathbf{V}\left({\hat{\beta }}_{7},{\hat{\beta }}_{9}\right)$ denotes the covariance matrix between the parameters corrected by the MSRC method. All simulations and analyses were performed using R 4.2.1 (R Foundation for Statistical Computing).

## Results

Table 1 presents the results of the regression coefficients, nuisance parameters, and type I error rates obtained for the two correction methods under the three intervention scenarios. The point estimates of the coefficients obtained from the GLM are extremely small-biased in all scenarios. For the variance ${\sigma }_{\alpha }^{2}$, the proposed MSRC does not change its estimate. Both methods use the estimator ${\hat{\sigma }}_{UB}^{2}$, with its point estimate very close to the true values when the sample size exceeds 180 (Supplemental Fig. S1A). For the autocorrelation parameter ${\rho }_{\alpha }$, the estimator ${\widehat{\rho }}_{UB}$ tends to underestimate as the autocorrelation coefficient increases (Supplemental Fig. S1B). Although this underestimation is relatively small, it still leads to an inflation of the type I error rate when the autocorrelation coefficient is high (≥ 0.6) (Supplemental Fig. S2). In contrast, the new estimator ${\widehat{\rho }}_{MSRC}$ is slightly overestimated (Supplemental Fig. S1C). When the sample size reaches 340, the type I error rate is controlled well in all simulation settings and is not overly conservative due to the slight overestimation of ${\widehat{\rho }}_{MSRC}$. Similar results are obtained for other settings for sample sizes, intercepts and linear trends (Supplemental Fig. S3).

**Table 1:** Point estimates and type I error rates for UB-corrected method and MSRC method

*N*	$\boldsymbol{\sigma}_{\boldsymbol{\alpha}}^{\boldsymbol{2}}$	$\boldsymbol{\rho }_{\boldsymbol{\alpha }}$	Level_bias	Trend_bias	$\boldsymbol{\sigma }_{\boldsymbol{UB}}^{\boldsymbol{2}}$^a^	UB-corrected				MSRC			
						$\boldsymbol{\rho}_{\boldsymbol{UB}}$^a^	Type I error rate			$\boldsymbol{\rho}_{\boldsymbol{MSRC}}$^a^	Type I error rate		
							Level change	Trend change	Both change		Level change	Trend change	Both change
20	0.5	0.2	0.005	-0.007	0.461	0.190	0.088	0.092	0.106	0.190	0.088	0.092	0.106
		0.4	0.018	0.059	0.399	0.259	0.114	0.130	0.159	0.259	0.114	0.130	0.159
		0.6	0.004	0.006	0.306	0.341	0.153	0.203	0.237	0.341	0.153	0.203	0.237
		0.8	0.005	-0.024	0.175	0.415	0.168	0.255	0.292	0.415	0.168	0.255	0.292
	1	0.2	0.021	-0.070	0.918	0.171	0.078	0.084	0.094	0.171	0.078	0.084	0.094
		0.4	0.023	0.022	0.789	0.242	0.114	0.135	0.153	0.242	0.114	0.135	0.153
		0.6	0.015	-0.138	0.623	0.315	0.152	0.202	0.233	0.315	0.152	0.202	0.233
		0.8	0.030	-0.076	0.356	0.392	0.167	0.278	0.308	0.391	0.167	0.278	0.308
180	0.5	0.2	0.001	-0.022	0.503	0.200	0.049	0.052	0.050	0.215	0.048	0.051	0.049
		0.4	0.005	-0.022	0.501	0.387	0.055	0.059	0.063	0.409	0.052	0.057	0.060
		0.6	0.004	-0.006	0.503	0.581	0.068	0.068	0.076	0.619	0.060	0.060	0.066
		0.8	0.007	-0.017	0.489	0.766	0.088	0.095	0.112	0.809	0.068	0.069	0.078
	1	0.2	0.007	0.009	1.003	0.206	0.052	0.050	0.051	0.228	0.050	0.048	0.049
		0.4	0.009	-0.019	1.005	0.397	0.053	0.052	0.057	0.421	0.050	0.050	0.055
		0.6	0.011	-0.016	1.011	0.586	0.070	0.064	0.073	0.626	0.060	0.057	0.065
		0.8	0.017	-0.006	0.993	0.767	0.089	0.101	0.113	0.808	0.068	0.079	0.086
340	0.5	0.2	0.000	0.007	0.500	0.199	0.052	0.052	0.056	0.215	0.050	0.051	0.054
		0.4	0.006	-0.007	0.501	0.397	0.053	0.056	0.060	0.430	0.048	0.051	0.055
		0.6	0.002	-0.008	0.503	0.591	0.058	0.060	0.065	0.643	0.043	0.044	0.046
		0.8	0.003	0.000	0.500	0.783	0.074	0.075	0.086	0.825	0.051	0.050	0.052
	1	0.2	-0.004	0.009	1.000	0.204	0.054	0.050	0.054	0.232	0.053	0.048	0.052
		0.4	0.002	-0.014	1.000	0.402	0.053	0.056	0.056	0.440	0.048	0.051	0.050
		0.6	-0.002	0.004	1.001	0.594	0.062	0.063	0.070	0.649	0.046	0.048	0.051
		0.8	0.017	-0.001	1.011	0.786	0.074	0.074	0.086	0.827	0.051	0.049	0.053

*UB-corrected* Unbiased correction, *MSRC* Maximum significant *ρ* correction

^a^In order to compare with the true values of the nuisance parameters, the estimators ${\hat{\sigma }}_{w,UB}^{2}$, ${\widehat{\rho }}_{w,UB}$, and ${\widehat{\rho }}_{w,MSRC}$for the process ${w}_{t}$ were all transformed into estimators ${\hat{\sigma }}_{UB}^{2}$, ${\widehat{\rho }}_{UB}$, and ${\widehat{\rho }}_{MSRC}$ corresponding to the latent process ${\alpha }_{t}$ by an exponential function

**Fig. 1:**
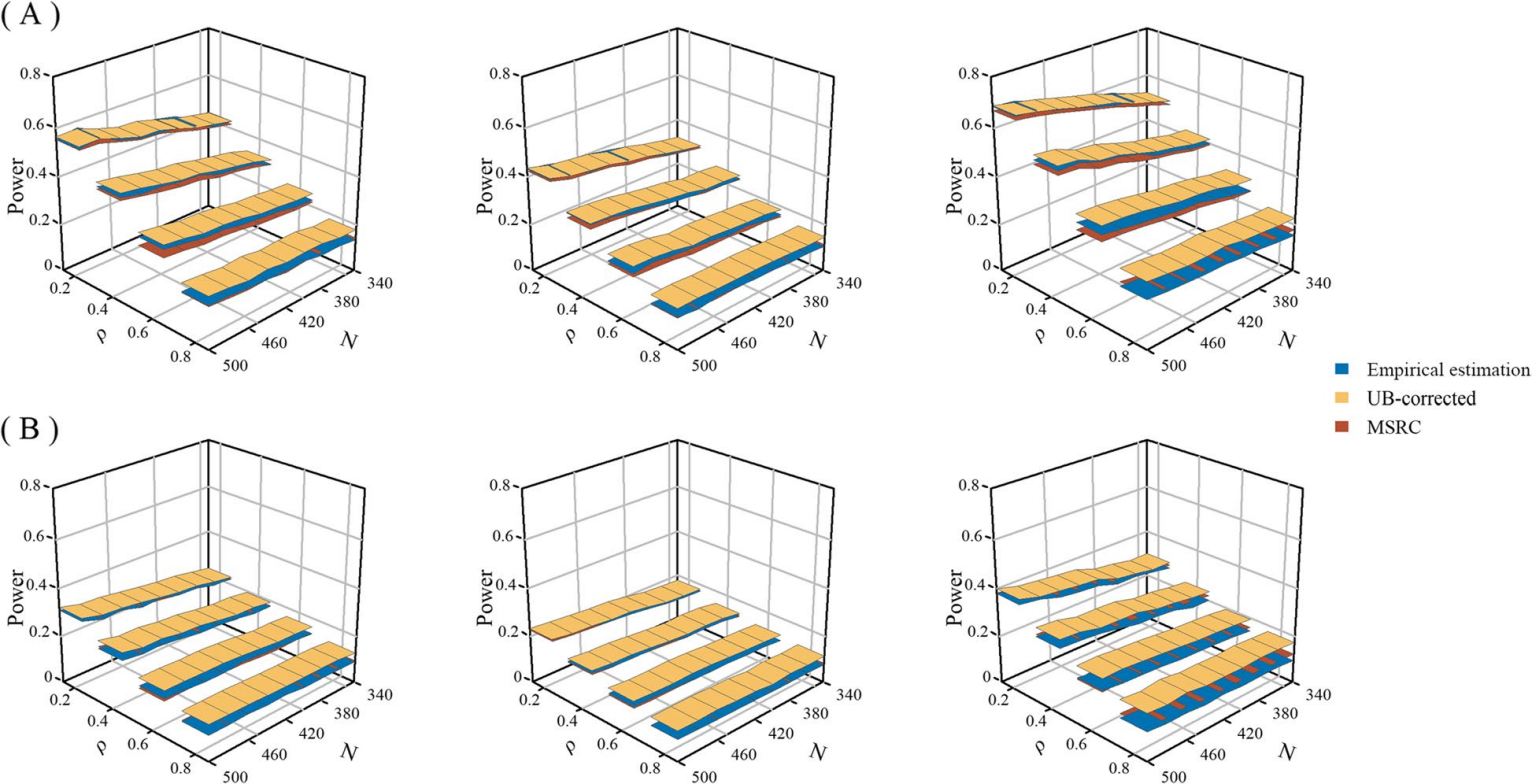
Statistical power under three intervention scenarios. UB-corrected: unbiased correction; MSRC: maximum significant *ρ* correction. The different colored bands indicate estimation methods. Panel (**A**) ${\sigma }_{\alpha }^{2}=0.5$; and Panel (**B**) ${\sigma }_{\alpha }^{2}=1$, and from left to right for the scenarios of level change ($\varvec{\beta }={\left(\text{1,1},\text{0.4,0}\right)}^{T}$), trend change (${\varvec{\beta }=\left(\text{1,1},\text{0,1.2}\right)}^{T}$), and both level and trend change (${\varvec{\beta }=\left(\text{1,1},\text{0.4,1.2}\right)}^{T}$)

Figure 1 shows the comparison of statistical power between the two correction methods and the empirical estimation. First, the trend of power estimated by these three methods is entirely consistent; specifically, it increases with increasing sample size and effect size and decreasing autocorrelation and variance of the latent process (Supplemental Fig. S4). Comparing the two correction methods, MSRC consistently exhibits lower power than UB-corrected in all simulation settings. The difference between them is negligible at small autocorrelation (≤ 0.4), but increases as the autocorrelation rises further. The power obtained by both correction methods is closer to the empirical power when the autocorrelation coefficient ${\rho }_{\alpha }$ is small (≤ 0.4). As ${\rho }_{\alpha }$ further increases, the power of MSRC is also similar to the empirical power. Furthermore, this finding still held across different sample sizes, effect sizes, and variances of the latent process.

The sensitivity analyses showed that the type I error rate remained uncontrolled when using a maximum order less than 5 (e.g., 3), and using a higher order over 5 (e.g., 7) produced slightly conservative results with a reducing power (Supplemental Tables S1 and S2).

**Fig. 2:**
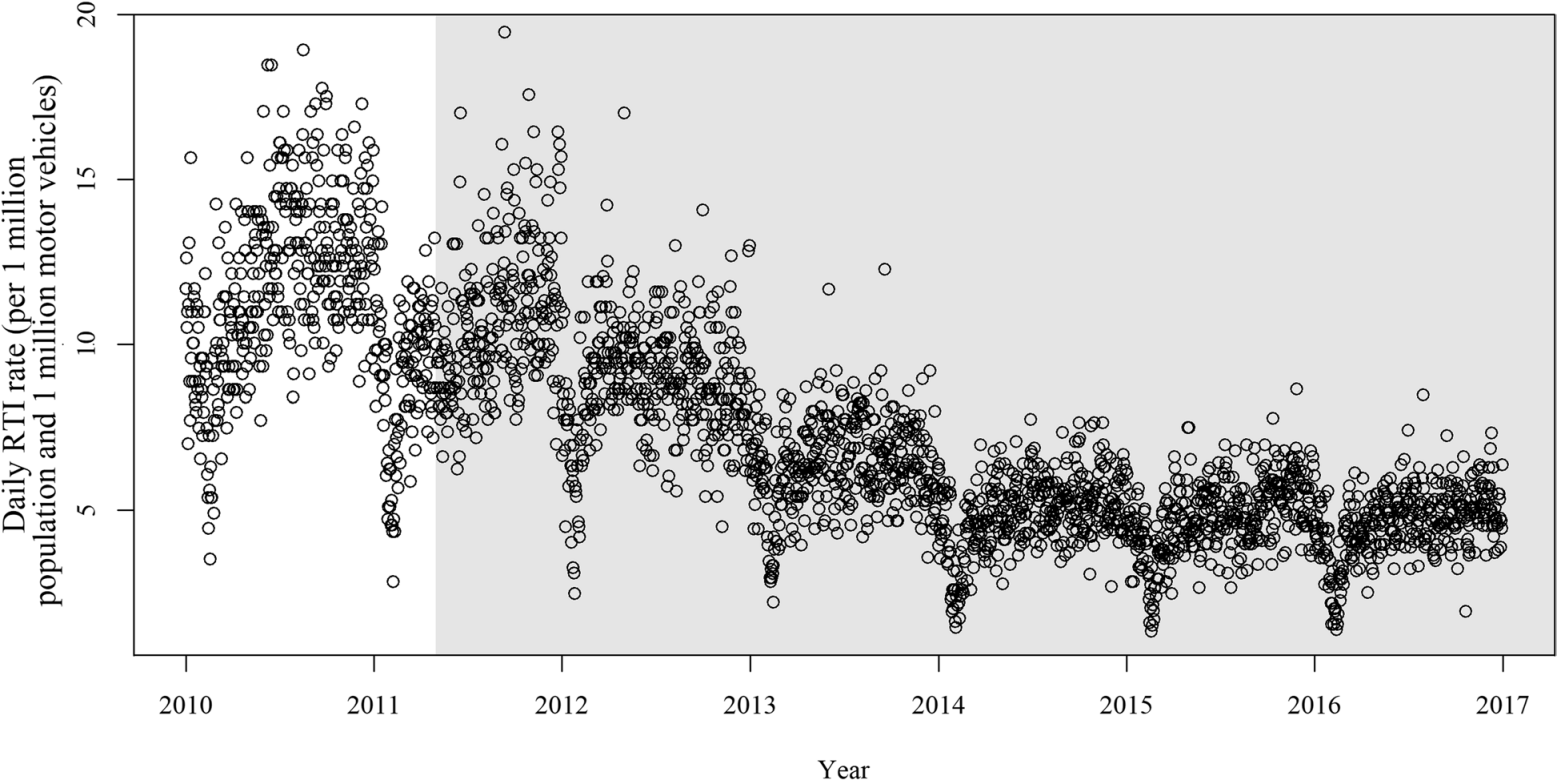
The incidence of daily road traffic injuries in Shenzhen, China, 2010–2016. RTI, road traffic injuries. The gray shadow shows the post-intervention period for drink driving interventions implemented since May 2011

**Fig. 3:**
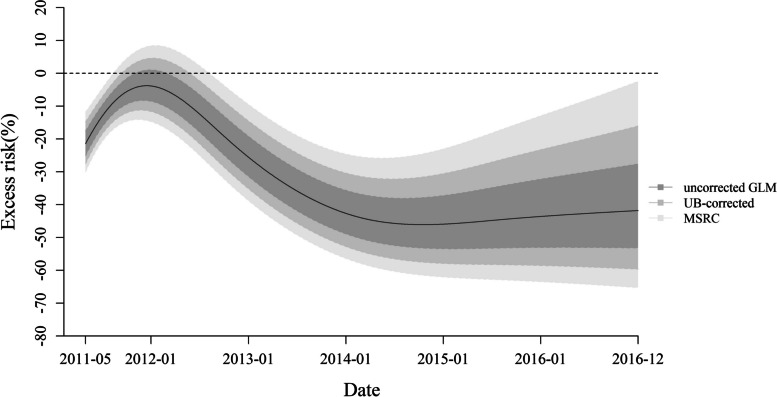
Excess risks of road traffic injuries over time estimated by three methods. GLM, generalized linear model; UB-corrected: unbiased correction; MSRC: maximum significant *ρ* correction

Figure 2 shows that the daily incidence of RTIs was relatively stable before the intervention and gradually decreased since May 2011. The partial autocorrelation function plot of residuals of the conventional GLM showed significant autocorrelation (Supplemental Fig. S5). Consequently, we further used the UB-corrected and MSRC methods (${\widehat{\rho }}_{UB}=0.379$ vs. ${\widehat{\rho }}_{MSRC}=0.704$). Three methods produced the same point estimations of the intervention effect, showing a weak or even non-significant effect at the beginning of the intervention and a rapid reduction in the risk of the incidence of RTIs since 2012, but the intervention effects gradually plateaued after 2014 (Fig. 3). Compared to the uncorrected GLM and UB-corrected, the MSRC had a wider confidence interval (CI), with an ER of -8.34% (95% CI, -20.51 to 5.69%), -45.07% (95% CI, -59.30% to -25.86%) and − 42.94% (95% CI, -64.00% to -9.56%) at 1, 3 and 5 years after the intervention, respectively (Table 2).

**Table 2:** Excess risks of road traffic injuries attributable to drunk driving intervention by three methods

	Excess risk% (95% CI)		
	May 01, 2012	May 01, 2014	May 01, 2016
Uncorrected GLM	-8.34 (-13.64 to -2.72)	-45.07 (-51.59 to -37.66)	-42.94 (-53.04 to -30.67)
UB-corrected	-8.34 (-17.12 to 1.37)	-45.07 (-55.59 to -32.05)	-42.94 (-58.85 to -20.88)
MSRC	-8.34 (-20.51 to 5.69)	-45.07 (-59.30 to -25.86)	-42.94 (-64.00 to -9.56)

*CI* Confidence interval, *GLM *Generalized linear model, *UB-corrected *Unbiased correction, *MSRC *Maximum significant *ρ* correction

## Discussion

In the realm of public health, autocorrelation in count time series is very common, posing challenges in model estimation and resulting in underestimation of SE and subsequent incorrect conclusions. However, despite the longstanding interest in this issue, the current body of literature lacks methods that can accurately estimate SE and well control the type I error rates. To fill this significant gap, we propose a novel alternative approach, known as the MSRC method, for modelling parameter-driven autocorrelated count time series with a well-controlled type I error rate. In addition, we applied this method to evaluate the effectiveness of drunk driving intervention on the incidence of RTIs in Shenzhen, China.

The autocorrelation of the latent process is particularly noteworthy in parameter-driven time series models with count data, as it directly affects the control of the type I error rate. For the UB-corrected method, the type I error rate remains within an acceptable range when the autocorrelation coefficient is small (${\rho }_{\alpha }$≤0.4). However, the type I error rate is inflated as the autocorrelation coefficient increases further. On the other hand, the MSRC method effectively controls the type I error rate across different levels of autocorrelation when the sample size approaches 340. Furthermore, notably, the autocorrelation function of the counting process is usually dominated by that of the latent process. This implies that significant autocorrelation present in the latent process may be masked when little or no autocorrelation is observed during the count time series [10]. Therefore, if it is challenging to determine the significance of the autocorrelation of the latent process, the MSRC method, which ensures robust control of the type I error rate may be a preferable choice.

Ye et al. (2022) [14] highlighted that the type I error rate is inflated when the coefficient $\beta_{t}$ of the pre-intervention trend term is not equal to 0. However, in practical scenarios, it is highly unlikely to have no trend before intervention. This may explain why the UB-corrected method still inflates type I error rates even under large sample sizes when $\beta_{t} \ne 0$ is set in our study. In contrast, the MSRC method demonstrates superior control over type I error rates in the presence of a pre-intervention trend, making it a more effective and reasonable choice for practical applications.

Many studies evaluating interventions rely on annual or monthly data with only a few dozen or even several time points available [27, 28]. However, our study highlights that the type I error rate is far from being controlled under a small sample size. The MSRC method proposed in this study demonstrates a well-controlled type I error rate when the sample size reaches approximately 340, corresponding to nearly 29-year monthly data, which is very difficult to collect in practice. Therefore, collecting data at a finer time scale (e.g., weekly or daily) may be a better choice, but it is still necessary to consider the number of events occurring at each time point. The sample size *n* considered in this study refers to the length of the time series, without explicitly specifying the number of events per time point. The true values of the regression coefficients were chosen to ensure that the expected events at each time point in all simulation settings were between 2 and 14, mainly to prevent the problem of zero inflation and power being all close to 1. Consequently, it is not advisable to obtain a longer time series by choosing a more precise time scale that would result in an excessive number of zero counts.

The commonly used methods for evaluating the effects of a public health intervention include the difference-in-difference (DID) method and ITS analysis. For example, Liang et al. (2008) [29] conducted a DID analysis to assess the intervention effect of zero-tolerance drunk driving regulation for drivers under 21 years in the United States, with a control group of contemporaneous college students aged 22–24 years. However, DID requires a homogenous parallel control, which is often very challenging since a public health intervention is generally implemented in the whole population. Moreover, as a non-randomized trial, the confounding factors reflecting between-group differences are not easy to be observed or controlled. In practice, most studies often use self-control to compare changes in outcomes before and after the intervention. Therefore, the ITS method proves to be a better choice, especially when the tricky issue of series autocorrelation was addressed in this study.

In summary, our study has three strengths. First, for parameter-driven autocorrelated time series with count data, the new MSRC method provides a simple implementation of asymptotic covariance estimation without the need for extensive computation to approximate the high-dimensional integration of the full likelihood function, which is attractive in practice. Second, the type I error rate of the proposed method is acceptable and robust. Moreover, the power of the new method closely approximates that of UB-corrected when the autocorrelation is small (≤ 0.4). As the autocorrelation coefficient increases further, the power obtained from the new method becomes more accurate, while the higher power of UB-corrected is most likely due to the inflated type I error rate. Therefore, the MSRC method is very competitive for parameter-driven autocorrelated time series with count data. Third, we evaluated the nonlinear time-varying effect of the drunk driving intervention on RTIs based on daily data and corrected the autocorrelation in the data to avoid false-positive conclusions.

However, there are several limitations. First, we only focused on the most common Poisson distribution for count data. Future extensions to negative binomial distributions and even the entire exponential distribution family are necessary to develop a unified modelling framework for parameter-driven GLMs. Second, the MSRC method performs well only for a large data set with sample sizes over 340. It is necessary to improve the parameter estimation method for small sample sizes in the future to enhance the practical application scenarios. Third, some count time series may have excessive zeros due to the rare occurrence of events of interest. Hence, it is valuable to develop a zero inflation model within this framework. Fourth, the time-varying population size and the number of motor vehicles were controlled in the real-data analysis. However, we have not considered vehicle type and road quality, which may have an impact on the incidence of RTIs, due to the unavailability of relevant data.

## Conclusions

This study proposed a MSRC method for modelling parameter-driven autocorrelated time series of count data, which solved the issue of underestimation of the asymptotic covariance matrix inherent in the classical UB-corrected method. The Monte Carlo simulation justified that the new estimator had good finite sample performance with a well-controlled type I error rate and efficient statistical power. Using the MSRC method to correct the covariance matrix in the presence of autocorrelation, the real analysis revealed that drunk driving regulations can significantly reduce the incidence of RTIs. This method provides a good modelling strategy for parameter-driven autocorrelated time series of count data and correct effect estimation in the field of public health intervention evaluation.

### Supplementary Information

**Supplementary Material 1.**

## Data Availability

All simulation data and analysis codes relevant to the study are uploaded as supplementary information. However, the road traffic injuries data used in the real data analysis were obtained from the Shenzhen pre-hospital care center. We have no right to make the original data public. Restrictions apply to the availability of these data, and so the data are not publicly available. Data are however available from the authors upon reasonable request and with permission of Shenzhen pre-hospital care center.

## Abbreviations

CI: Confidence interval
DID: Difference-in-difference
ER: Excess risk
GLM: Generalized linear model
ITS: Interrupted time-series
MSRC: Maximum significant *ρ* correction
RTIs: Road traffic injuries
SE: Standard error
UB-corrected: Unbiased correction
