# Supplementary material for: A novel correction method for modelling parameter-driven autocorrelated time series with count outcome
Source: BMC Public Health. 2024 Mar 27;24:901. doi: 10.1186/s12889-024-18382-4 (PMC10967065; doi:10.1186/s12889-024-18382-4)
Supplement: Supplementary file 1 — Supplementary Material 1. [file 12889_2024_18382_MOESM1_ESM.docx]

**Supplemental Code S1** Simulation code for parameter-driven autocorrelated time series with count outcome based on interrupt time series.

library(MASS)

#------------------------------------------------------------------------------------------------

# nsmp: The total sample size (the number of time points)

# pre_nsmp: The number of time points before the intervention

# bet_inter: The coefficient of intercept term

# bet_t: The coefficient of time

# bet_X: The coefficient of intervention variable

# bet_Xt: The coefficient of the interaction term between time and intervention variable

# rho: Autocorrelation coefficient

# sigma_mu2: Variance

# greatmod: Formula of independent variables

# sim: Number of simulations

#------------------------------------------------------------------------------------------------

ITSsim_para<-function(nsmp,pre_nsmp,bet_inter,bet_t,bet_X,bet_Xt,rho,sigma_mu2,greatmod,sim){

set.seed(202305)

#==================================functions

correctcov<-function(data,beta,sigma,rho){

n<-dim(data)[1]

m<-dim(data)[2]

SigmaI<-matrix(0,m,m)

SigmaII<-matrix(0,m,m)

for (p in 1:n){

dummy_SigmaI <- (data[p,]%*%t(data[p,]))*c(exp(t(data[p,])%*%beta))

SigmaI<-SigmaI+dummy_SigmaI

}

for (p in 1:n){

for (q in 1:n){

dummy_SigmaII <- (data[p,]%*%t(data[q,]))*c(exp((t(data[p,])+t(data[q,]))%*%beta))*(sigma*(rho^abs(p-q)))

SigmaII<-SigmaII+dummy_SigmaII

}}

varbeta<-solve(SigmaI)+solve(SigmaI)%*%SigmaII%*%solve(SigmaI)

sebeta<-sqrt(diag(varbeta))

list(varbeta=varbeta,sebeta=sebeta)

}

rhofun<-function(yy,Xdata,beta){

nsmp<-length(yy)

GEEmu<-exp(Xdata%*%beta)

GEEsigma2<-sum((yy-GEEmu)^2-GEEmu)/sum(GEEmu^2)

if(GEEsigma2<0) {GEEsigma2<-1e-4}

num<-den<-rep(NA,nsmp-1)

for(m in 1:(nsmp-1)){

num[m]<-(yy[m]-GEEmu[m])*(yy[m+1]-GEEmu[m+1])

den[m]<-(GEEmu[m])*(GEEmu[m+1])

}

GEErho1<-sum(num,na.rm = T)/(GEEsigma2*sum(den))

if(GEErho1>1) {GEErho1<-0.99}

if(GEErho1<0) {GEErho1<-0}

list(GEEsigma=GEEsigma2,GEErho=GEErho1)

}

correctcovUB<-function(data,beta,sigma,rho,L=15){

n<-dim(data)[1]

m<-dim(data)[2]

SigmaI<-matrix(0,m,m)

SigmaII<-matrix(0,m,m)

mu<-exp(data%*%beta)

for (p in 1:n){

dummy_SigmaI <- (data[p,]%*%t(data[p,]))*mu[p]

SigmaI<-SigmaI+dummy_SigmaI

}

for (p in (-L):L){

for (q in max(1-p,1):min(n-p,n)){

dummy_SigmaII <- (data[q,]%*%t(data[q+p,]))*mu[q]*mu[q+p]*(sigma*(rho^abs(p)))

SigmaII<-SigmaII+dummy_SigmaII

}}

G<-solve(SigmaI)+solve(SigmaI)%*%SigmaII%*%solve(SigmaI)

return(G)

}

rhofunUB<-function(yy,Xdata,beta,sigmapre,rhopre){

nsmp<-length(yy)

## σUB

GEEmu<-exp(Xdata%*%beta)

G<-correctcovUB(Xdata,beta=beta,sigmapre,rhopre,L=15)

dummy1<-exp(-2*Xdata%*%G%*%t(Xdata))

dummy2<-exp(2*Xdata%*%G%*%t(Xdata))

dummy3<-exp(Xdata%*%G%*%t(Xdata)/2)

sigmaUB<-sum((yy-GEEmu)^2+GEEmu^2*diag(dummy1)*diag(dummy2-2*dummy3+1)-GEEmu)/sum(GEEmu^2*diag(dummy1))

if(is.na(sigmaUB) | sigmaUB<0) {sigmaUB<-1e-4}

## ρUB & ρMSRC

rhoUBvector<-rhoUBvectorC<-VrhoUBvector<-Prhovector<-NULL;rankUB<-1

while(rankUB>0 & rankUB<6){

g<-gamaUB1<-gamaUB2<-VrhoUB1<-VrhoUB2<-rep(NA,nsmp-rankUB)

for (k in 1:(nsmp-rankUB)){

g[k]<-exp(-(Xdata[k,]+Xdata[k+rankUB,])%*%G%*%(Xdata[k,]+Xdata[k+rankUB,])/2)

gamaUB1[k]<-GEEmu[k]*GEEmu[k+rankUB]*g[k]

gamaUB2[k]<-(yy[k]-GEEmu[k])*(yy[k+rankUB]-GEEmu[k+rankUB])+GEEmu[k]*GEEmu[k+rankUB]*g[k]*(1-diag(dummy3)[k]-diag(dummy3)[k+rankUB]+1/g[k])

VrhoUB1[k]<-GEEmu[k]*GEEmu[k+rankUB]

VrhoUB2[k]<-GEEmu[k]^2*GEEmu[k+rankUB]^2*(1+1/GEEmu[k]/sigmaUB)*(1+1/GEEmu[k+rankUB]/sigmaUB)

}

gamaUB<-sum(gamaUB2)/sum(gamaUB1)

rhoUB<-gamaUB/sigmaUB

if(is.na(rhoUB)) {rhoUB<-0}

if(rhoUB>1) {rhoUB<-0.99}

rhoUBvector<-rbind(rhoUBvector,rhoUB)

VrhoUB<-sum(VrhoUB2)/(sum(VrhoUB1)^2)

VrhoUBvector<-rbind(VrhoUBvector,VrhoUB)

Prho<-ifelse(( 2*(1-pnorm( abs(rhoUB/sqrt(VrhoUB)) ))<0.01 ),1,0)

Prhovector<-rbind(Prhovector,Prho)

if(rhoUB<0) {rhoUBvectorC<-rbind(rhoUBvectorC,NA)} else {rhoUBvectorC<-rbind(rhoUBvectorC,(rhoUB)^(1/rankUB))}

rankUB=rankUB+1

}

if(rhoUBvector[1]<0) {rhoUBvector[1]<-0}

rhoUBvectorC[which(is.na(rhoUBvectorC))]<-0

if (sum(Prhovector==1)==0) {order=1} else {order=c(which(Prhovector==1))}

list(GEEsigmaUB=sigmaUB,GEErhoUB=rhoUBvector[1],GEErhomax=max(rhoUBvectorC[order],na.rm = T),order=mean(order) )

}

##===========Generate data

X <-c(rep(0,pre_nsmp), rep(1,nsmp-pre_nsmp))

time <-c(1:nsmp)/nsmp

Xt <-X*(time*nsmp-pre_nsmp)/nsmp

Xtdata<-model.matrix( ~ time + X + Xt )

beta <-c(bet_inter, bet_t, bet_X, bet_Xt)

mu0 <-apply(Xtdata, 1, function(s){sum(s*beta)})

mu1 <-exp(mu0)

sigma_ep2<-sigma_mu2*(1-rho^2)

Xmatrix<-as.matrix(data.frame(inter=rep(1,nsmp),time=time,X=X,Xt=Xt))

pchi_2= qchisq(0.95, 2)

matrixy5<-NULL

bias_GLM5X<-NULL;bias_GLM5Xt<-NULL

powerX_UB<-NULL;powerXt_UB<-NULL;powerXXt_UB<-NULL

powerX_max<-NULL;powerXt_max<-NULL;powerXXt_max<-NULL

powerXXt_MC<-NULL

sigmavector5<-NULL;

rhoUBvector5<-NULL;rhomaxvector5<-NULL;ordervector<-NULL;

modwarn<-NULL

for(i in 1:sim){

u5<-rep(NA,nsmp+1);y5<-rep(NA,nsmp)

u5[1]<-rnorm(1,-sigma_mu2/2,sqrt(sigma_mu2))

for (j in 1:nsmp){

u5[j+1]<-rho*(u5[j]+sigma_mu2/2)+rnorm(1,0,sqrt(sigma_ep2))-sigma_mu2/2

y5[j]<-rpois(1,exp(mu0[j]+u5[j+1])) }

matrixy5<-rbind(matrixy5,y5)

mod_GLM5w<- tryCatch({ mod_GLM5<-eval(parse(text=paste( "glm(y5~",greatmod,",family=poisson)",sep=""))) },warning = function(w){1})

if (is.list(mod_GLM5w)){

bias_GLM5X<-cbind(bias_GLM5X,coef(mod_GLM5)[3])

bias_GLM5Xt<-cbind(bias_GLM5Xt,coef(mod_GLM5)[4])

dummy5<-rhofun(y5,Xmatrix,coef(mod_GLM5))

dummy5UB<-rhofunUB(y5,Xmatrix,coef(mod_GLM5),dummy5$GEEsigma,dummy5$GEErho)

sigmavector5<-cbind(sigmavector5,dummy5UB$GEEsigmaUB)

ordervector<-cbind(ordervector,dummy5UB$order)

#------------------------------unbiased correction

rhoUBvector5<-cbind(rhoUBvector5,dummy5UB$GEErhoUB)

vovUB5<-correctcov(Xmatrix,coef(mod_GLM5),dummy5UB$GEEsigmaUB,dummy5UB$GEErhoUB)

#Level change

countX_UB<-ifelse(( 2*(1-pnorm(abs(coef(mod_GLM5)[3]/vovUB5$sebeta[3] )))<0.05 ),1,0)

powerX_UB<-cbind(powerX_UB,countX_UB)

#Trend change

countXt_UB<-ifelse(( 2*(1-pnorm(abs(coef(mod_GLM5)[4]/vovUB5$sebeta[4] )))<0.05 ),1,0)

powerXt_UB<-cbind(powerXt_UB,countXt_UB)

#Both level and trend change

countXXt_UB<-ifelse( coef(mod_GLM5)[3:4]%*%solve(vovUB5$varbeta[3:4,3:4])%*%coef(mod_GLM5)[3:4]>pchi_2 ,1,0)

powerXXt_UB<-cbind(powerXXt_UB,countXXt_UB)

#------------------------------maximum significant ρ correction

rhomaxvector5<-cbind(rhomaxvector5,dummy5UB$GEErhomax)

vovmax5<-correctcov(Xmatrix,coef(mod_GLM5),dummy5UB$GEEsigmaUB,dummy5UB$GEErhomax)

#Level change

countX_max<-ifelse(( 2*(1-pnorm(abs(coef(mod_GLM5)[3]/vovmax5$sebeta[3] )))<0.05 ),1,0)

powerX_max<-cbind(powerX_max,countX_max)

#Trend change

countXt_max<-ifelse(( 2*(1-pnorm(abs(coef(mod_GLM5)[4]/vovmax5$sebeta[4] )))<0.05 ),1,0)

powerXt_max<-cbind(powerXt_max,countXt_max)

#Both level and trend change

countXXt_max<-ifelse( coef(mod_GLM5)[3:4]%*%solve(vovmax5$varbeta[3:4,3:4])%*%coef(mod_GLM5)[3:4]>pchi_2 ,1,0)

powerXXt_max<-cbind(powerXXt_max,countXXt_max)

modwarn<-cbind(modwarn,0) } else {modwarn<-cbind(modwarn,1)}

}

#------------------------------empirical estimation

#Level change

powerX_MC<-ifelse(( 2*(1-pnorm(abs(bias_GLM5X/sd(bias_GLM5X) )))<0.05 ),1,0)

#Trend change

powerXt_MC<-ifelse(( 2*(1-pnorm(abs(bias_GLM5Xt/sd(bias_GLM5Xt) )))<0.05 ),1,0)

#Both level and trend change

matrixMC<-matrix(c(as.vector(bias_GLM5X),as.vector(bias_GLM5Xt)),sim,2)

for (j in 1:sim){

countXXt_MC<-ifelse( (matrixMC[j,])%*%solve( cov(matrixMC) )%*%(matrix(matrixMC[j,],2,1))>pchi_2 ,1,0)

powerXXt_MC<-cbind(powerXXt_MC,countXXt_MC)

}

##===========Results

NSum5<-colSums(matrixy5[which(modwarn!=1),])/(sim-sum(modwarn))

## point estimation & Type I error rate & Power

Tvalue1<-bet_X

Tvalue2<-bet_Xt

pointest5<-data.frame(N=nsmp,beta_int=bet_inter,beta_t=bet_t,beta_X=bet_X,beta_Xt=bet_Xt,rho=rho,sigma2=sigma_mu2,

biasX=mean(bias_GLM5X)-Tvalue1,seX=sd(bias_GLM5X),MSEX=mean((bias_GLM5X-Tvalue1)^2),

biasXt=mean(bias_GLM5Xt)-Tvalue2,seXt=sd(bias_GLM5Xt),MSEXt=mean((bias_GLM5Xt-Tvalue2)^2),

alphaUBX=sum(powerX_UB)/(sim-sum(modwarn)),alphaUBXt=sum(powerXt_UB)/(sim-sum(modwarn)),alphaUBXXt=sum(powerXXt_UB)/(sim-sum(modwarn)),

alphamaxX=sum(powerX_max)/(sim-sum(modwarn)),alphamaxXt=sum(powerXt_max)/(sim-sum(modwarn)),alphamaxXXt=sum(powerXXt_max)/(sim-sum(modwarn)),

alphaMCX=sum(powerX_MC)/(sim-sum(modwarn)),alphaMCXt=sum(powerXt_MC)/(sim-sum(modwarn)),alphaMCXXt=sum(powerXXt_MC)/(sim-sum(modwarn)),

rhoUBE=mean(rhoUBvector5),rhomaxE=mean(rhomaxvector5),sigmaE=mean(sigmavector5),order=mean(ordervector),

before_N=mean(NSum5[1:(nsmp/2)]),after_N=mean(NSum5[(nsmp/2+1):(nsmp)]) , modwarn=sum(modwarn))

list(mu0=mu0,yy5=matrixy5,pointest5=round(pointest5,3),modwarn=modwarn)

}

## Example

ITSsim_para(nsmp=340,pre_nsmp=170,bet_inter=1,bet_t=1,bet_X=0,bet_Xt=0,rho=0.2,sigma_mu2=0.5,greatmod="time+X+Xt",sim=10000)

**Supplemental Table S1** Type I error rates of maximum significant *ρ* correction method at different orders.

| *N* | $\sigma_{\alpha}^{2}$ | $\rho_{\alpha}$ | $\sigma_{UB}^{2}$^a^ | 3 order | | | | 7 order | | | |
| --- | --- | --- | --- | --- | --- | --- | --- | --- | --- | --- | --- |
|  |  |  |  | $\rho_{MSRC}$^a^ | Level  change | Trend  change | Both change | $\rho_{MSRC}$^a^ | Level  change | Trend  change | Both change |
| 20 | 0.5 | 0.2 | 0.461 | 0.190 | 0.088 | 0.092 | 0.106 | 0.190 | 0.088 | 0.092 | 0.106 |
|  |  | 0.4 | 0.399 | 0.259 | 0.114 | 0.130 | 0.159 | 0.259 | 0.114 | 0.130 | 0.159 |
|  |  | 0.6 | 0.306 | 0.341 | 0.153 | 0.203 | 0.237 | 0.341 | 0.153 | 0.204 | 0.237 |
|  |  | 0.8 | 0.175 | 0.415 | 0.168 | 0.255 | 0.292 | 0.415 | 0.168 | 0.255 | 0.292 |
|  | 1 | 0.2 | 0.918 | 0.171 | 0.078 | 0.084 | 0.094 | 0.172 | 0.078 | 0.084 | 0.094 |
|  |  | 0.4 | 0.789 | 0.242 | 0.114 | 0.135 | 0.153 | 0.242 | 0.114 | 0.135 | 0.153 |
|  |  | 0.6 | 0.623 | 0.315 | 0.152 | 0.202 | 0.233 | 0.314 | 0.152 | 0.202 | 0.234 |
|  |  | 0.8 | 0.356 | 0.392 | 0.167 | 0.278 | 0.308 | 0.391 | 0.167 | 0.278 | 0.308 |
| 180 | 0.5 | 0.2 | 0.503 | 0.208 | 0.049 | 0.051 | 0.049 | 0.223 | 0.048 | 0.050 | 0.049 |
|  |  | 0.4 | 0.501 | 0.403 | 0.053 | 0.058 | 0.061 | 0.415 | 0.051 | 0.057 | 0.060 |
|  |  | 0.6 | 0.503 | 0.613 | 0.061 | 0.060 | 0.067 | 0.623 | 0.059 | 0.059 | 0.065 |
|  |  | 0.8 | 0.489 | 0.801 | 0.070 | 0.074 | 0.084 | 0.812 | 0.066 | 0.068 | 0.076 |
|  | 1 | 0.2 | 1.003 | 0.217 | 0.051 | 0.048 | 0.050 | 0.240 | 0.049 | 0.047 | 0.048 |
|  |  | 0.4 | 1.005 | 0.415 | 0.050 | 0.050 | 0.055 | 0.429 | 0.049 | 0.050 | 0.054 |
|  |  | 0.6 | 1.011 | 0.619 | 0.062 | 0.057 | 0.065 | 0.630 | 0.060 | 0.056 | 0.064 |
|  |  | 0.8 | 0.993 | 0.800 | 0.072 | 0.082 | 0.089 | 0.811 | 0.067 | 0.079 | 0.085 |
| 340 | 0.5 | 0.2 | 0.500 | 0.208 | 0.051 | 0.052 | 0.055 | 0.226 | 0.049 | 0.050 | 0.053 |
|  |  | 0.4 | 0.501 | 0.423 | 0.049 | 0.052 | 0.055 | 0.436 | 0.048 | 0.050 | 0.054 |
|  |  | 0.6 | 0.503 | 0.633 | 0.045 | 0.046 | 0.048 | 0.647 | 0.043 | 0.043 | 0.045 |
|  |  | 0.8 | 0.500 | 0.814 | 0.056 | 0.056 | 0.060 | 0.830 | 0.049 | 0.047 | 0.049 |
|  | 1 | 0.2 | 1.000 | 0.218 | 0.053 | 0.049 | 0.053 | 0.248 | 0.052 | 0.047 | 0.052 |
|  |  | 0.4 | 1.000 | 0.430 | 0.050 | 0.052 | 0.051 | 0.450 | 0.048 | 0.050 | 0.049 |
|  |  | 0.6 | 1.001 | 0.639 | 0.048 | 0.049 | 0.052 | 0.655 | 0.045 | 0.047 | 0.050 |
|  |  | 0.8 | 1.011 | 0.816 | 0.057 | 0.056 | 0.062 | 0.833 | 0.048 | 0.048 | 0.051 |

^a^In order to compare with the true values of the nuisance parameters, the estimators $\hat{\sigma}_{w,UB}^{2}$ and $\hat{\rho}_{w,MSRC}$ for the process $w_{t}$ were all transformed into estimators $\hat{\sigma}_{UB}^{2}$ and $\hat{\rho}_{MSRC}$ corresponding to the latent process $\alpha_{t}$ by an exponential function.

**Supplemental Table S2** Statistical power of maximum significant *ρ* correction method at different orders.

| *N* | $\sigma_{\alpha}^{2}$ | $\rho_{\alpha}$ | 5 order | | | 7 order | | |
| --- | --- | --- | --- | --- | --- | --- | --- | --- |
|  |  |  | Level change | Trend  change | Both change | Level  change | Trend  change | Both change |
| 340 | 0.5 | 0.2 | 0.420 | 0.313 | 0.505 | 0.415 | 0.310 | 0.500 |
|  |  | 0.4 | 0.309 | 0.244 | 0.396 | 0.304 | 0.241 | 0.390 |
|  |  | 0.6 | 0.218 | 0.153 | 0.266 | 0.213 | 0.151 | 0.263 |
|  |  | 0.8 | 0.142 | 0.113 | 0.177 | 0.135 | 0.109 | 0.168 |
|  | 1 | 0.2 | 0.225 | 0.171 | 0.290 | 0.222 | 0.169 | 0.285 |
|  |  | 0.4 | 0.177 | 0.136 | 0.222 | 0.174 | 0.134 | 0.217 |
|  |  | 0.6 | 0.129 | 0.101 | 0.159 | 0.126 | 0.100 | 0.156 |
|  |  | 0.8 | 0.094 | 0.083 | 0.122 | 0.090 | 0.080 | 0.116 |
| 400 | 0.5 | 0.2 | 0.475 | 0.360 | 0.580 | 0.469 | 0.355 | 0.572 |
|  |  | 0.4 | 0.351 | 0.267 | 0.432 | 0.346 | 0.264 | 0.426 |
|  |  | 0.6 | 0.232 | 0.176 | 0.299 | 0.226 | 0.174 | 0.294 |
|  |  | 0.8 | 0.156 | 0.121 | 0.189 | 0.148 | 0.116 | 0.178 |
|  | 1 | 0.2 | 0.254 | 0.192 | 0.314 | 0.248 | 0.188 | 0.309 |
|  |  | 0.4 | 0.198 | 0.144 | 0.245 | 0.194 | 0.141 | 0.241 |
|  |  | 0.6 | 0.140 | 0.114 | 0.173 | 0.136 | 0.112 | 0.169 |
|  |  | 0.8 | 0.101 | 0.081 | 0.129 | 0.095 | 0.077 | 0.122 |
| 500 | 0.5 | 0.2 | 0.552 | 0.426 | 0.670 | 0.543 | 0.421 | 0.660 |
|  |  | 0.4 | 0.425 | 0.309 | 0.524 | 0.419 | 0.304 | 0.517 |
|  |  | 0.6 | 0.278 | 0.201 | 0.341 | 0.271 | 0.197 | 0.333 |
|  |  | 0.8 | 0.174 | 0.128 | 0.218 | 0.164 | 0.122 | 0.206 |
|  | 1 | 0.2 | 0.311 | 0.226 | 0.385 | 0.304 | 0.221 | 0.377 |
|  |  | 0.4 | 0.238 | 0.172 | 0.289 | 0.233 | 0.170 | 0.284 |
|  |  | 0.6 | 0.150 | 0.122 | 0.188 | 0.147 | 0.120 | 0.184 |
|  |  | 0.8 | 0.110 | 0.089 | 0.137 | 0.103 | 0.084 | 0.129 |


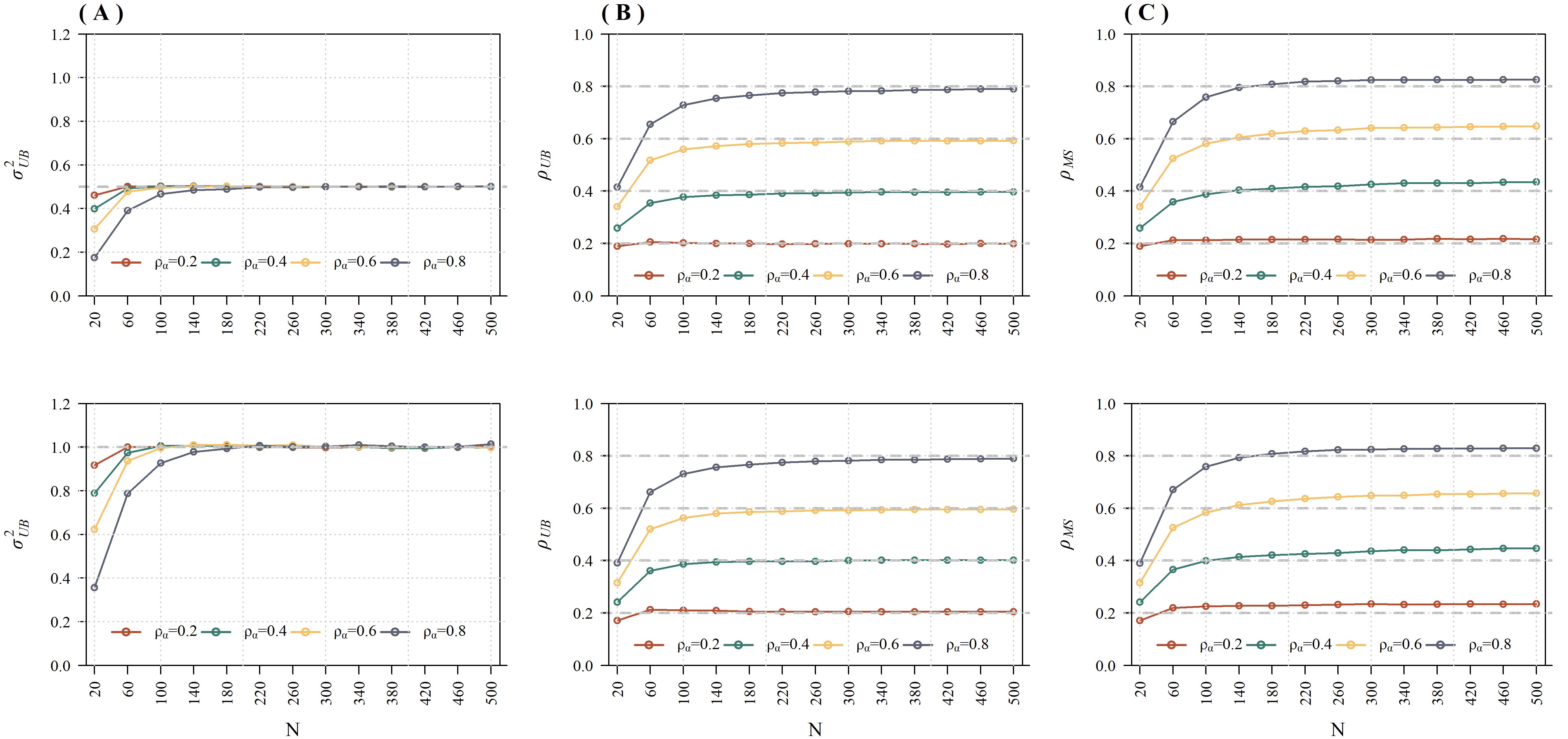


**Supplemental Fig. S1** Estimation of the variance and autocorrelation coefficients of nuisance parameters. The different colored lines indicate different values of $\rho_{\alpha}$. Panel (A) shows the estimates of $\hat{\sigma}_{UB}^{2}$, and panels (B) and (C) represent the estimates of $\hat{\rho}_{UB}$ and $\hat{\rho}_{MSRC}$, respectively. The top half represents the scenario with $\sigma_{\alpha}^{2}=0.5$ and the bottom half represents the scenario with $\sigma_{\alpha}^{2}=1$.


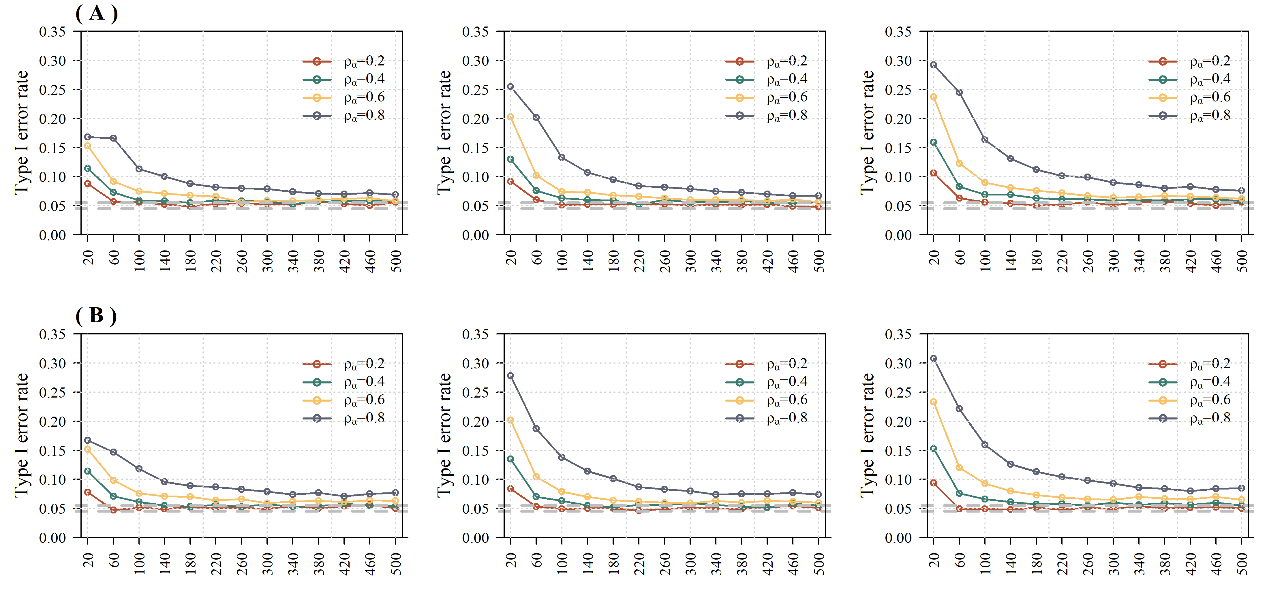


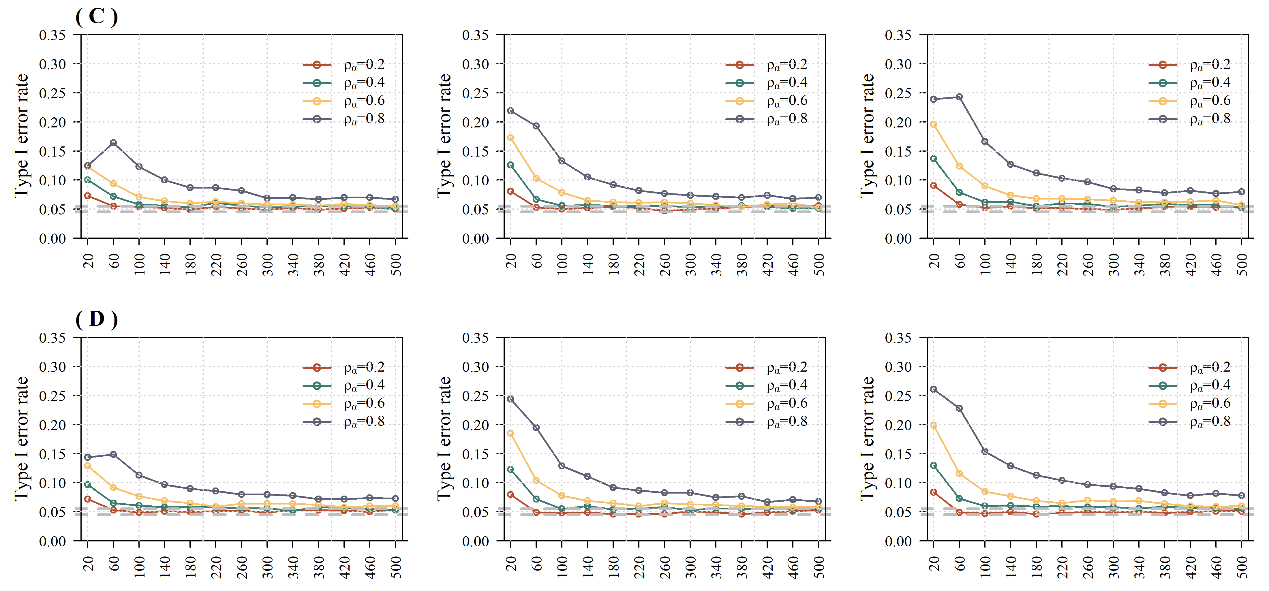


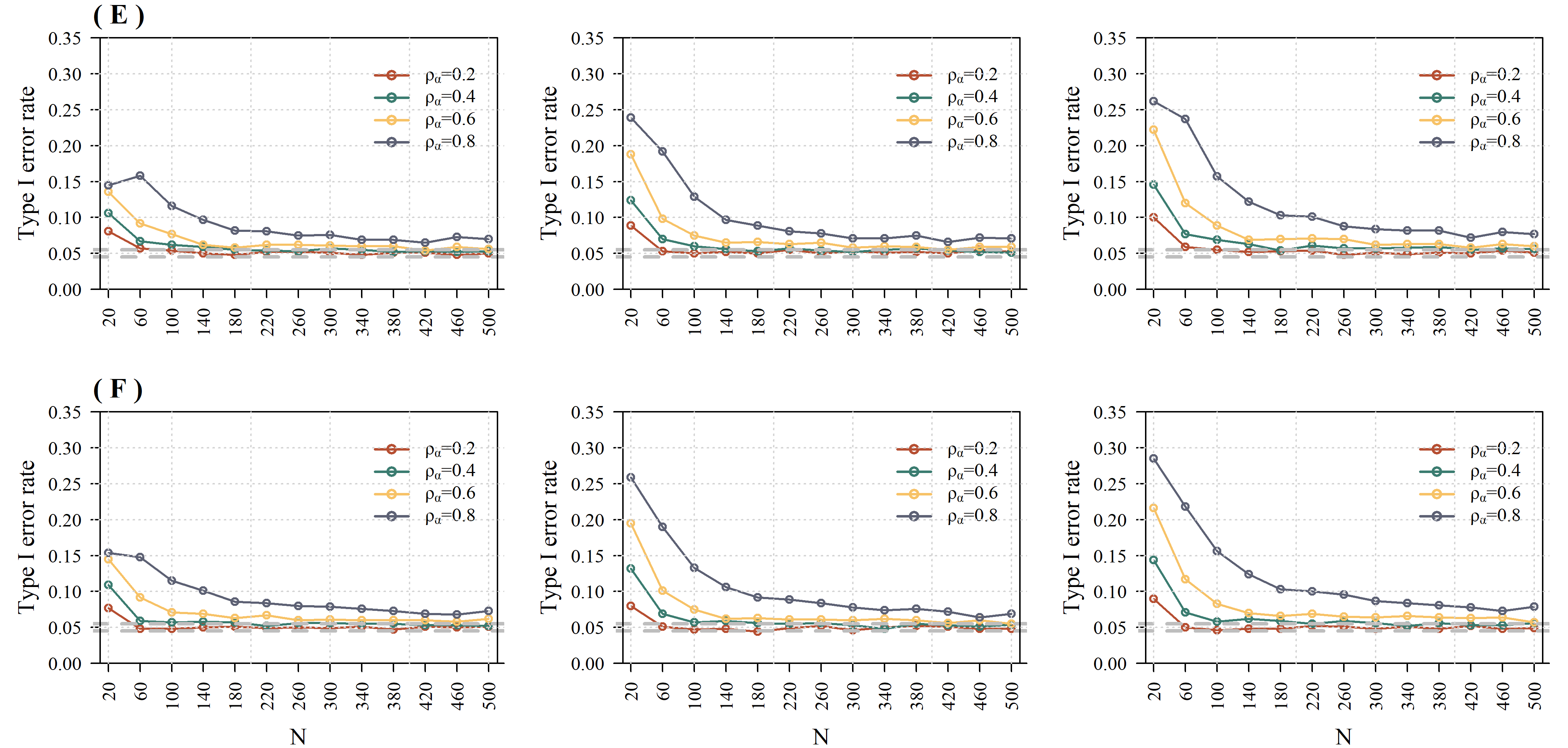


**Supplemental Fig. S2** Type I error rate of unbiased correction method under three intervention scenarios. From left to right for the scenarios of level change, trend change, and both level and trend change. The different colored lines indicate different values of $\rho_{\alpha}$. Panel (A) $\sigma_{\alpha}^{2}=0.5$ and ${\boldsymbol{\beta}=\left( 1,1,0,0 \right)}^{T}$, Panel (B) $\sigma_{\alpha}^{2}=1$ and ${\boldsymbol{\beta}=\left( 1,1,0,0 \right)}^{T}$, Panel (C) $\sigma_{\alpha}^{2}=0.5$ and ${\boldsymbol{\beta}=\left( 0.5,1,0,0 \right)}^{T}$, Panel (D) $\sigma_{\alpha}^{2}=1$ and ${\boldsymbol{\beta}=\left( 0.5,1,0,0 \right)}^{T}$, Panel (E) $\sigma_{\alpha}^{2}=0.5$ and ${\boldsymbol{\beta}=\left( 1,0.5,0,0 \right)}^{T}$, Panel (F) $\sigma_{\alpha}^{2}=1$ and ${\boldsymbol{\beta}=\left( 1,0.5,0,0 \right)}^{T}$.

**
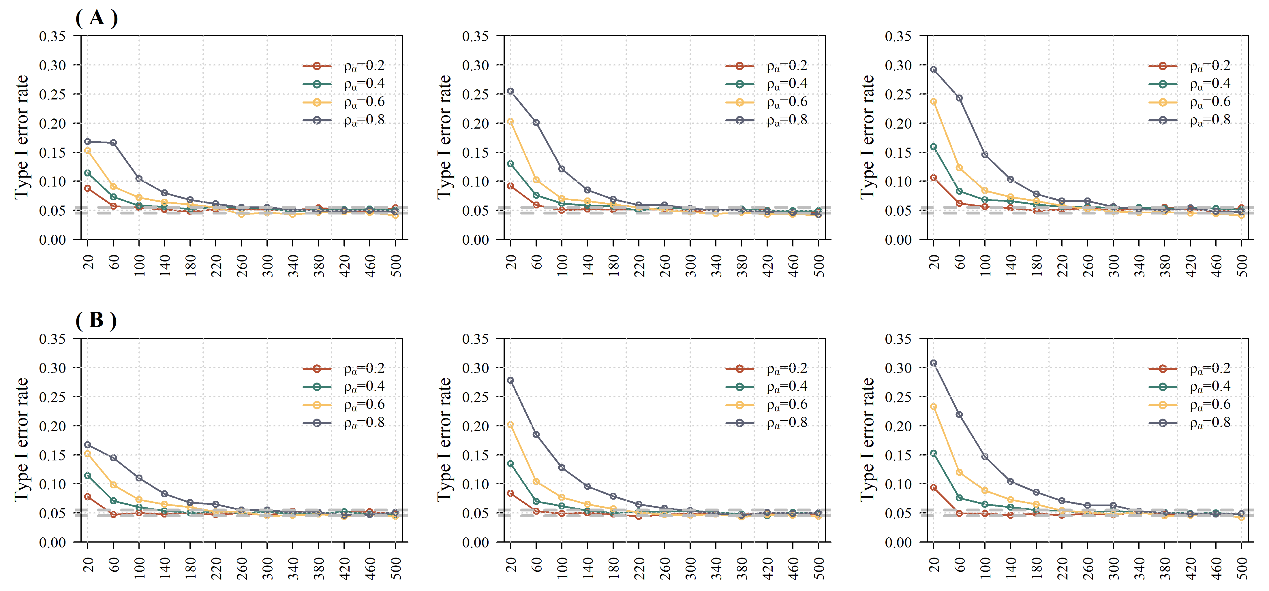
**

**
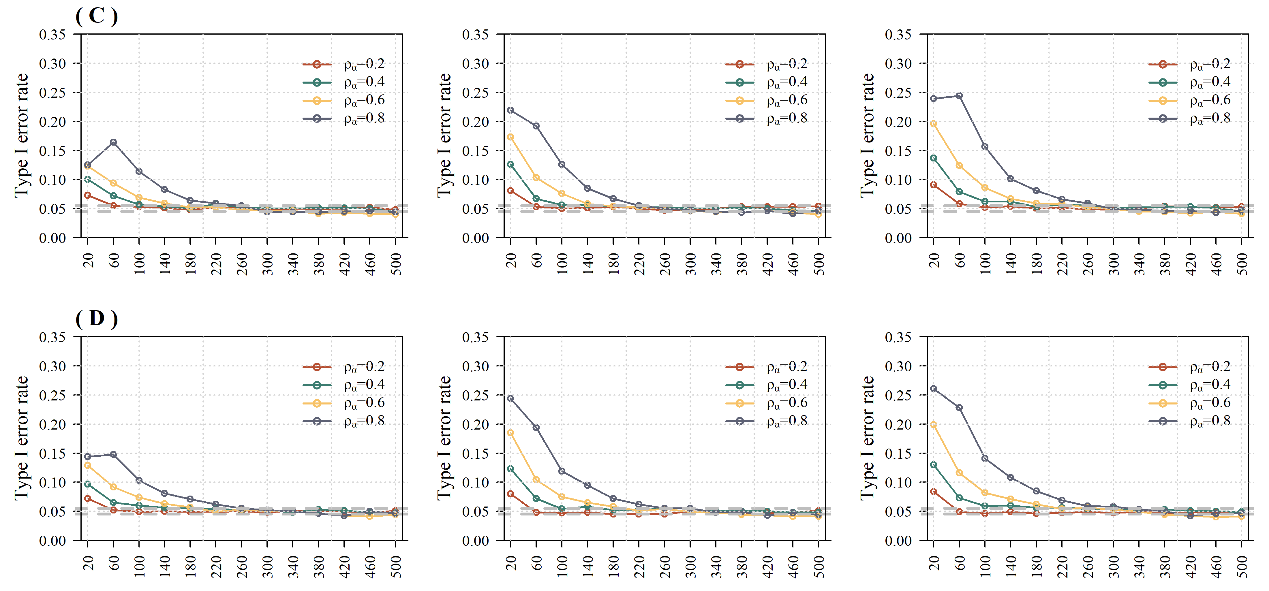
**


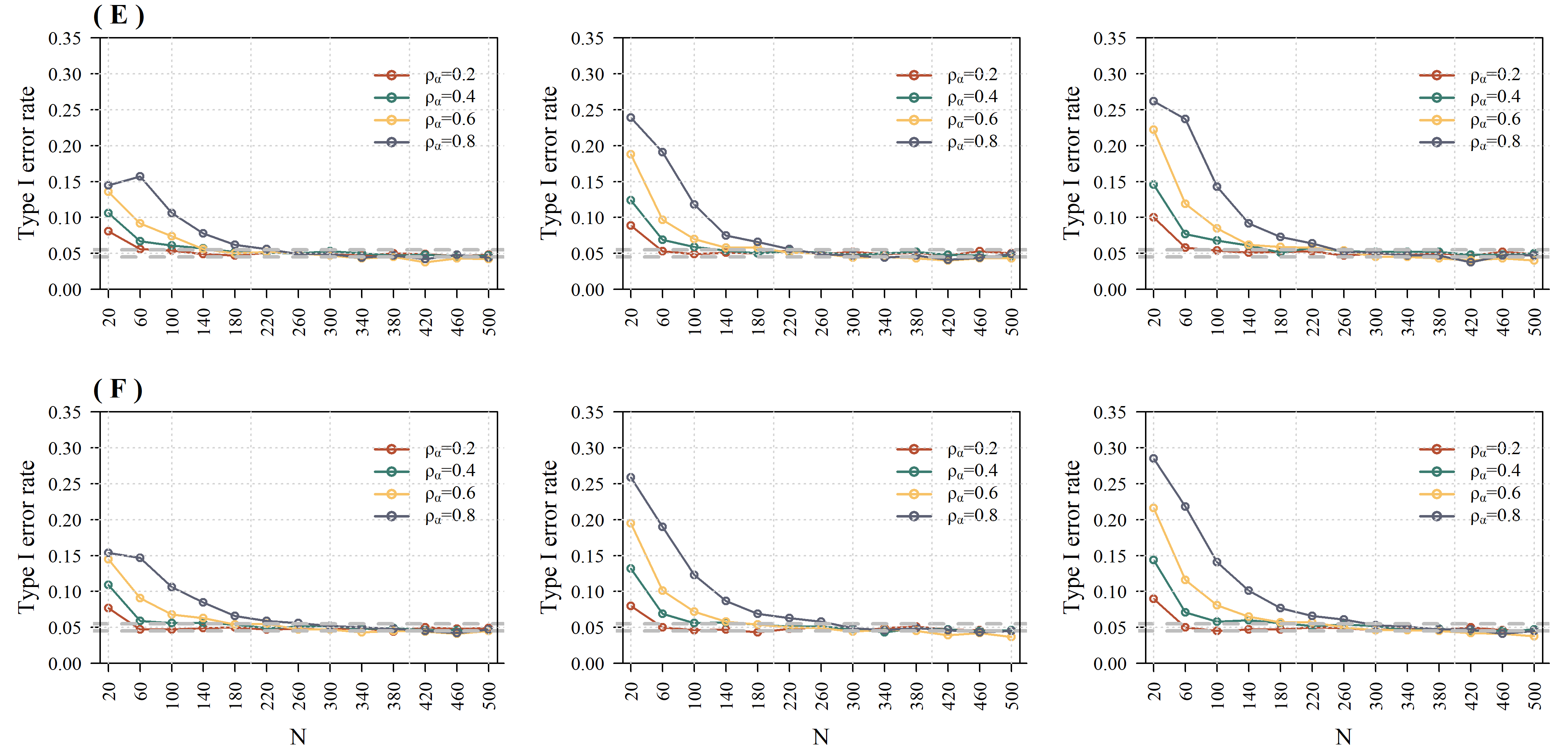


**Supplemental Fig. S3** Type I error rate of maximum significant *ρ* correction method under three intervention scenarios. From left to right for the scenarios of level change, trend change, and both level and trend change. The different colored lines indicate different values of $\rho_{\alpha}$. Panel (A) $\sigma_{\alpha}^{2}=0.5$ and ${\boldsymbol{\beta}=\left( 1,1,0,0 \right)}^{T}$, Panel (B) $\sigma_{\alpha}^{2}=1$ and ${\boldsymbol{\beta}=\left( 1,1,0,0 \right)}^{T}$, Panel (C) $\sigma_{\alpha}^{2}=0.5$ and ${\boldsymbol{\beta}=\left( 0.5,1,0,0 \right)}^{T}$, Panel (D) $\sigma_{\alpha}^{2}=1$ and ${\boldsymbol{\beta}=\left( 0.5,1,0,0 \right)}^{T}$, Panel (E) $\sigma_{\alpha}^{2}=0.5$ and ${\boldsymbol{\beta}=\left( 1,0.5,0,0 \right)}^{T}$, Panel (F) $\sigma_{\alpha}^{2}=1$ and ${\boldsymbol{\beta}=\left( 1,0.5,0,0 \right)}^{T}$.

**
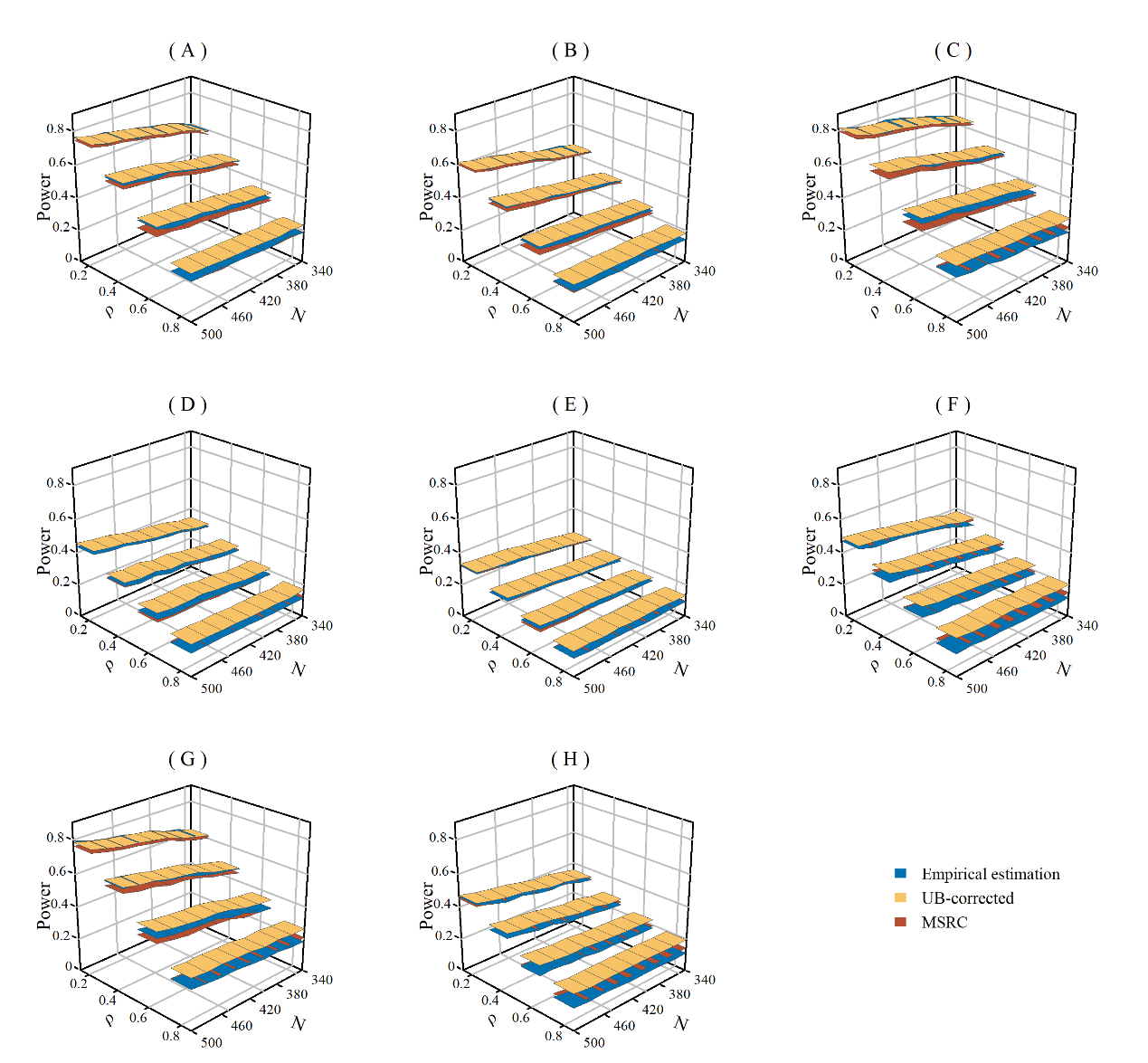
**

**Supplemental Fig. S4** Effect of effect sizes on statistical power under three intervention scenarios. UB-corrected: unbiased correction; MSRC: maximum significant *ρ* correction. The different colored bands indicate estimation methods. Panel (A) $\sigma_{\alpha}^{2}=0.5$; and level change ($\boldsymbol{\beta}=\left( 1,1,0.5,0 \right)^{T}$), Panel (B) $\sigma_{\alpha}^{2}=0.5$; and trend change (${\boldsymbol{\beta}=\left( 1,1,0,1.5 \right)}^{T}$), Panel (C) $\sigma_{\alpha}^{2}=0.5$; and both level and trend change (${\boldsymbol{\beta}=\left( 1,1,0.5,1.2 \right)}^{T}$), Panel (D) $\sigma_{\alpha}^{2}=1$; and level change ($\boldsymbol{\beta}=\left( 1,1,0.5,0 \right)^{T}$), Panel (E) $\sigma_{\alpha}^{2}=1$; and trend change (${\boldsymbol{\beta}=\left( 1,1,0,1.5 \right)}^{T}$), Panel (F) $\sigma_{\alpha}^{2}=1$; and both level and trend change (${\boldsymbol{\beta}=\left( 1,1,0.5,1.2 \right)}^{T}$), Panel (G) $\sigma_{\alpha}^{2}=0.5$; and both level and trend change (${\boldsymbol{\beta}=\left( 1,1,0.4,1.5 \right)}^{T}$), Panel (H) $\sigma_{\alpha}^{2}=1$; and both level and trend change (${\boldsymbol{\beta}=\left( 1,1,0.4,1.5 \right)}^{T}$).


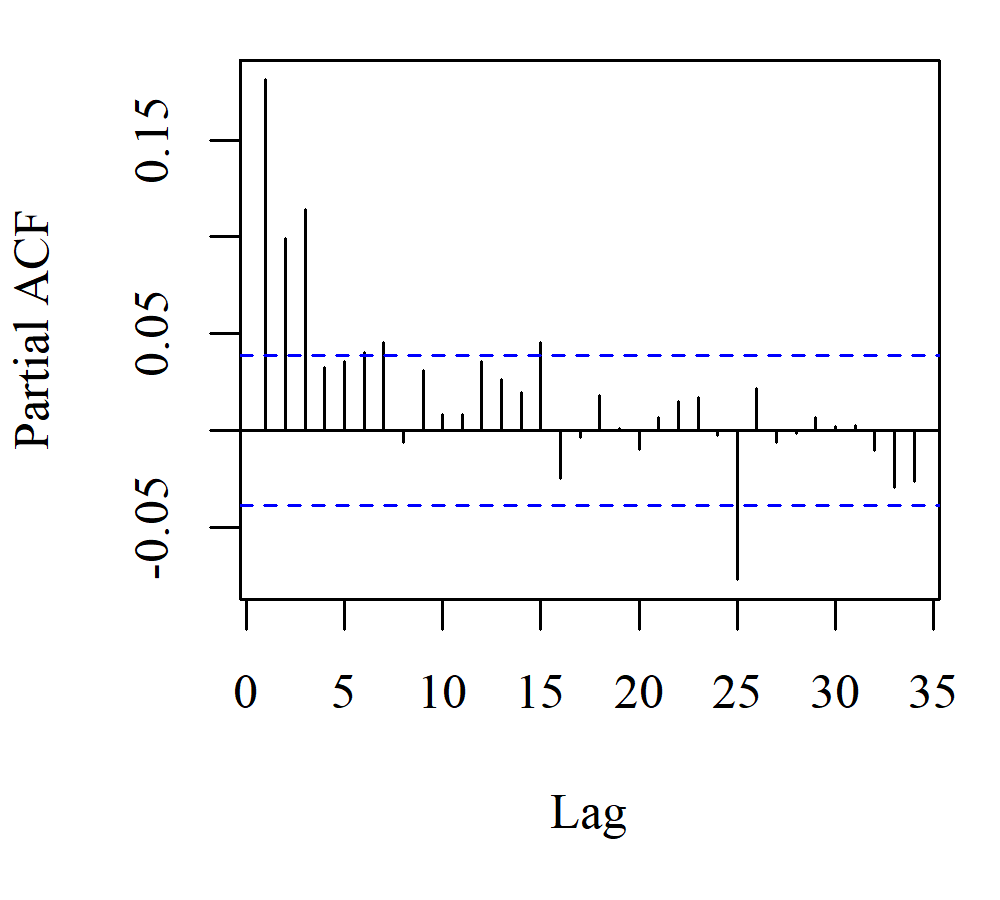


**Supplemental Fig. S5** The partial autocorrelation coefficient function (PACF) plots of the residuals of uncorrected Generalized Linear Model.
